# The Internal Organization of Mycobacterial Partition Assembly: Does the DNA Wrap a Protein Core?

**DOI:** 10.1371/journal.pone.0052690

**Published:** 2012-12-20

**Authors:** Shuo Qian, Rebecca Dean, Volker S. Urban, Barnali N. Chaudhuri

**Affiliations:** 1 Center for Structural Molecular Biology, Biology and Soft Matter Division, Oak Ridge National Laboratory, Oak Ridge, Tennessee, United States of America; 2 Hauptman Woodward Institute, Buffalo, New York, United States of America; 3 Department of Structural Biology, State University of New York, Buffalo, New York, United States of America; University of South Florida College of Medicine, United States of America

## Abstract

Before cell division in many bacteria, the ParBs spread on a large segment of DNA encompassing the origin-proximal *parS* site(s) to form the partition assembly that participates in chromosome segregation. Little is known about the structural organization of chromosomal partition assembly. We report solution X-ray and neutron scattering data characterizing the size parameters and internal organization of a nucleoprotein assembly formed by the mycobacterial chromosomal ParB and a 120-meric DNA containing a *parS*-encompassing region from the mycobacterial genome. The cross-sectional radii of gyration and linear mass density describing the rod-like ParB-DNA assembly were determined from solution scattering. A “DNA outside, protein inside” mode of partition assembly organization consistent with the neutron scattering hydrogen/deuterium contrast variation data is discussed. In this organization, the high scattering DNA is positioned towards the outer region of the partition assembly. The new results presented here provide a basis for understanding how ParBs organize the *parS*-proximal chromosome, thus setting the stage for further interactions with the DNA condensins, the origin tethering factors and the ParA.

## Introduction

An active segregation ensures the equivalent DNA distribution amongst daughter cells, which is a crucial cellular process in bacteria [1]–[2]. The bacterial partitioning cassette (ParABS) or the segrosome participates in the segregation of many plasmids and chromosomes [1]–[4]. More than 2/3^rd^ of the sequenced bacterial genomes harbor genes for the segrosome components [5]–[6]. Based on the nature of the motor protein involved, the segrosomes are classified into three major types [1]–[2]: type I (involving Walker A Cytoskeletal ATPase or WACA, [7]), type II (involving actin-like ParM ATPase) and type III (involving GTPase). The chromosomal segrosomes exclusively form a subtype of the type I family [1]–[2], that typically contains a motor protein (ParA, which is a WACA), a centromeric DNA binding protein (ParB) and a set of centromere-like DNA sequence(s) near the origin of replication (*parS*). Chromosomal ParBs spread on the DNA template in the *parS*-adjacent region [8]–[10] to form the partition assembly, which is a higher-order nucleo-protein complex of unknown nature.

The partition assembly recruits a number of proteins, each with a significant role in the bacterial cell cycle. An interaction between the partition assembly and the ParA is required for the ParABS-mediated DNA movement in many bacteria [3]–[4], [11]–[14]. In addition, the partition assembly interacts with the SMC proteins or DNA condensins in *Bacillus subtilis* and *Streptococcus pneumoniae* for accurate chromosome segregation [15]–[17]. In *Caulobacter crescentus*, the origin-proximal ParB-assembly associates with a polymeric cell-pole organization factor, PopZ, which tethers the origin region to the cell pole [18]. MipZ, which is the essential cell-division site selection protein in *C. crescentus*, interacts with the ParB-DNA assembly at the cell pole while synchronizing the DNA segregation with cell division [19]. The partition assembly directly interacts with the apical growth factor DivIVA in actinobacteria for tethering the chromosomal origin to the cell pole [20]. The precise natures of the interactions between multiple proteins and the partition assembly are not known.

Chromosomal *parS* is typically a conserved, 14 residue long, palindromic ‘GTTTCACGTGAAAC’ sequence. Sequence analysis identified potential *parS* sites in a number of bacteria [5]–[6]. A large variability in their numbers, positions and spacing between them has been reported [6]. Recently it has been shown that the *parS* site plays a crucial role in determining the overall genome orientation in *C. crescentus* [21]. The *parS* site appears to nucleate a condensed chromosome conformation, probably due to the assembling of ParBs and DNA condensins [21]. Although much progress have been made on understanding the plasmid-based partition assemblies of type I and type II categories [22], how the chromosomal ParBs organize the *parS*-encompassing region for segregation is yet to be determined.

The ParABS plays a key role in the mycobacterial cell cycle progression [23]–[25]. However, little is understood about the organization and function of the ParABS in pathogenic mycobacteria. Previously we described the solution organizations of chromosomal ParB (tbParB) from *Mycobacterium tuberculosis* (MTB) in the apo form [26] and in a complex with the *parS* DNA [27]. Here, we report the internal organization of the partition assembly formed by tbParB and a 120-meric DNA from the MTB genome containing a *parS* site using solution X-ray and neutron scattering (SAXS/SANS) with hydrogen/deuterium (H/D) contrast variation. Our data suggests that the DNA wraps around a protein core in the mycobacterial partition assembly, which serves to organize the DNA in a more compact form. The biological relevance of our result is discussed.

## Materials and Methods

### Materials

The tbParB was expressed and purified as described previously [27]. All experiments were performed in buffer A (10–50 mM Tris. HCl pH 8, 150 mM NaCl and 10% glycerol), unless otherwise noted. A 120-meric DNA and its complementary DNA (henceforth referred to as D120, CTGCTGCAGCGCCGATGGGGGTGTCGAATTCTGTCGATGTTTCACGTGAAACATTCATCGTCGGATTGTGCGCGGCCTCAGGCGTCGGTGTCGGTGGTGTCATTTCCCGCTGGAATGGTT, the *parS* sequence is underlined) were chemically synthesized, gel purified and annealed by heating at 95 C followed by slow cooling (The Keck oligonucleotide synthesis facility, Yale University). The duplex D120 was incubated with purified tbParB, co-purified as a nucleoprotein complex using a size-exclusion column (Superdex200, GE healthcare, Milwaukee, Wisconsin) and concentrated prior to the scheduled solution scattering experiments.

### Small Angle X-ray Scattering (SAXS)

SAXS experiments on the tbParB-D120 complex were performed at the BL4-2 beamline at the Stanford Synchrotron Radiation Laboratory (SSRL, [28]–[29]), at an X-ray energy of ∼11 keV and 2.5 m sample-to-detector distance. Silver behenate was used as a standard calibration to convert detector pixel value to the X-ray scattering vector q in Å^−1^, defined as q = 4πsin(θ)/λ, where 2θ is the scattering angle and λ is the wavelength of the incident X-ray beam. All data were collected using the auto-sampler controlled by Blu-Ice [28]. The subsequent data reduction steps, such as radial integration, intensity scaling, frame-averaging and back-ground subtraction, were performed by the program SASTOOL [29].

### Solution Neutron Scattering (SANS) with H/D-contrast Variation

The tbParB-DNA complex at 1.9 mg/ml protein concentration was dialyzed in 10 mM Tris.HCl at pH 8.0, 10% glycerol and different amounts of D_2_O (Cambridge Isotope Laboratories, Inc, www.isotope.com) prior to neutron scattering data collection. Neutron scattering data were collected at the CG-3 Bio-SANS instrument [30] at the High Flux Isotope Reactor facility of Oak Ridge National Laboratory (ORNL). A complete set of data covering the momentum transfer range q from 0.007 Å^−1^ to 0.38 Å^−1^ were taken at two sample-to-detector distances 2.5 m and 6.8 m with wavelength λ = 6 Å and a spread 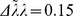. Samples were measured in the quartz “banjo” cells (Hellma USA, Plainview, NY) with 1 mm path length and 3–4 hours exposure time. Two dimensional data from the 192 by 192 pixels position-sensitive detector (ORDELA, Inc., Oak Ridge, TN) were corrected for the detector dark current, pixel sensitivity and then azimuthally averaged to produce the one dimensional scattering profile I(q) *versus* q, which was normalized to the incident neutron beam flux. Data reduction was performed by software developed at the neutron scattering facilities of ORNL. After merging the data from two sample-to-detector distances, the backgrounds from the buffer and quartz cell, as well as the incoherent scattering were subtracted from the scattering profiles. Experimental scattering intensity was converted to absolute neutron scattering cross-section per unit volume in units of cm^−1^ by using a calibration standard of known cross-section (Figure S1). The percentages of D_2_O in the H/D contrast measurement series (12.0%, 73.5% and 85.5%) were calculated using the neutron transmission data. Data analysis and size calculations were performed using MULCH [31] and the ATSAS suite of software including PRIMUS and GNOM [32]–[34].

## Results

### The Size of Partition Assembly in Solution

**Figure 1:**
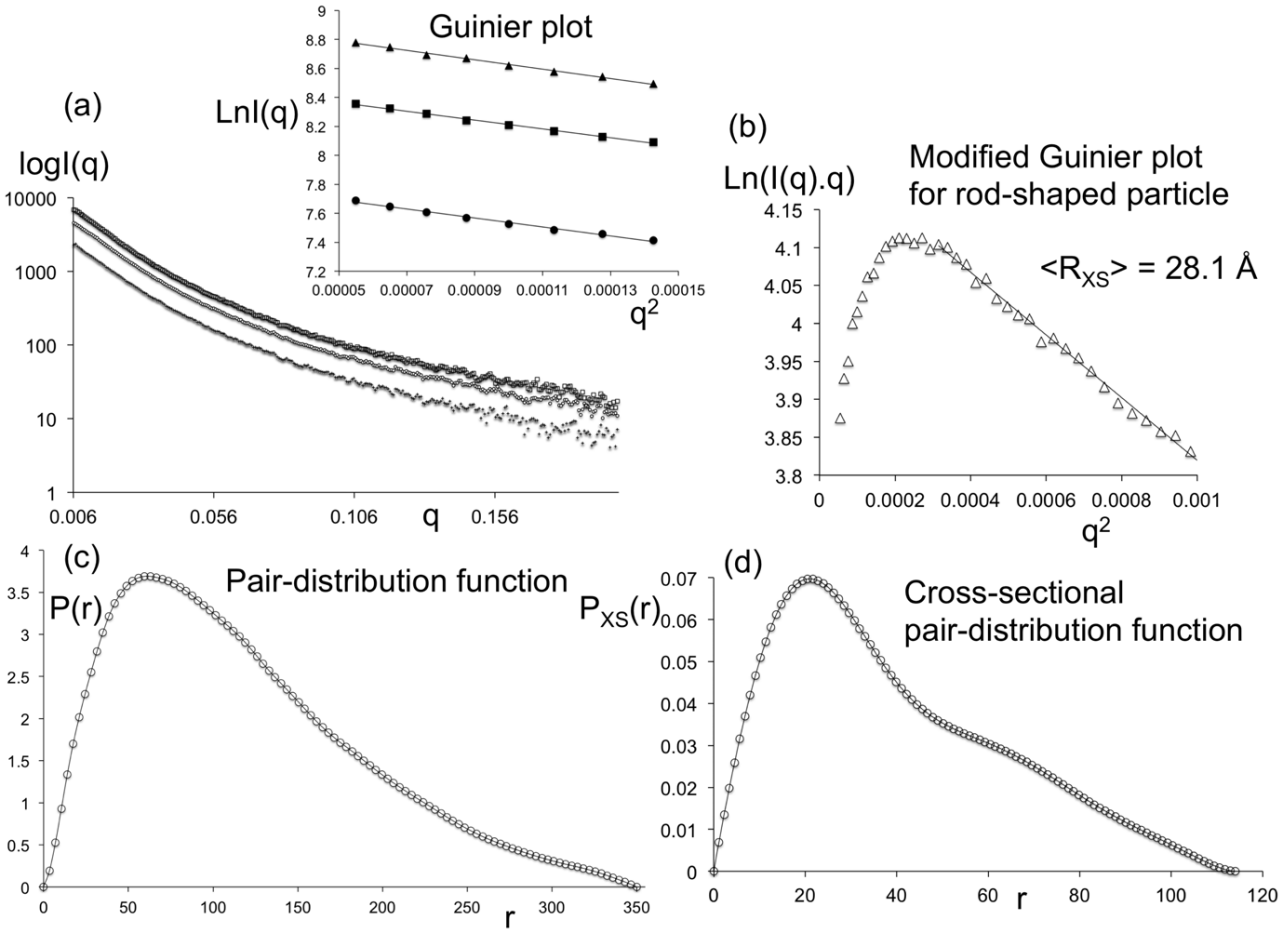
**The size of tbParB-D120 assembly derived from the SAXS data.** (a) Intensity (I) in arbitrary unit *versus* momentum transfer q in Å^−1^ are plotted at 3 different concentrations. The Guinier plot (lnI(q) *versus* q^2^, q in Å^−1^; 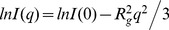) is shown in the inset. These and other graphs presented in this work are prepared using Excel® (Microsoft® corporation). A linear trend-line fitted to the data points is shown in each case. (b) The modified Guinier plot for rod-shaped particle (lnI(q)q *versus* q^2^, q in Å^−1^, 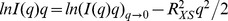) is shown. (c) The pair-distribution function P(r) *versus* pair-wise distance r in Å. The pair functions shown here and in the figure 5 were calculated with the following boundary conditions: P(r = 0) = 0 and P(r ≥ D_max_) = 0. (d) The cross-sectional pair-distribution function (P_XS_(r)) *versus* pair-wise distance r in Å.

The solution scattering technique was employed to characterize the size parameters of a complex between the tbParB and a 120-meric DNA containing a 14-meric *parS* site (tbParB-D120, Figure 1a). Unlike many other bacterial plasmids and chromosomes, *Mycobacteria* contains very few *parS* sites [6], [23]. Two 14-meric *parS* sites have been identified in the origin-proximal region of the MTB genome, which are separated by about 850 nucleotides. The 120-meric DNA contains sequences encompassing one *parS* site within the MTB chromosome (see Experimental Procedures section). The tbParB-D120 assembly formation was confirmed by electron microscopy (unpublished data). The average radius of gyration (R_g_) over three concentrations and the real-space R_g_ of the tbParB-D120, determined from the conventional Guinier plots and the pair-distribution function (P(r)), are 94.3 Å (q.R_g_ <1.2) and 96.5 Å, respectively (Figure 1a, inset). Averaged cross-sectional radius of gyration (R_xs_) of the tbParB-D120 was determined to be 28.1 Å from the slopes of the modified Guinier plots at three concentrations (Figure 1b) [35]. R_g_ and R_XS_ values determined at three concentrations were within 5% of the average. The maximum particle diameters from the P(r) and the cross-sectional pair distribution function (P_XS_(r)) were assigned to be 350 Å and 114 Å (Figures 1c and 1d). The diameter D and the length L of a uniform rod-shaped particle were estimated from the radii of gyrations (R_g_ and R_XS_), as follows:
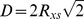


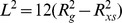



A length-to-diameter ratio larger than 3 is consistent with a rod-like shape of the tbParB-D120 assembly. The shape of modified Guinier plot and a left-skewed profile of the pair distribution function (Figure 1b–c) further supports a rod-like extended structure of the tbParB-D120 in solution.

### Stuhrmann Plot Suggests a “DNA Outside, Protein Inside” Organization of the Partition Assembly

**Figure 2:**
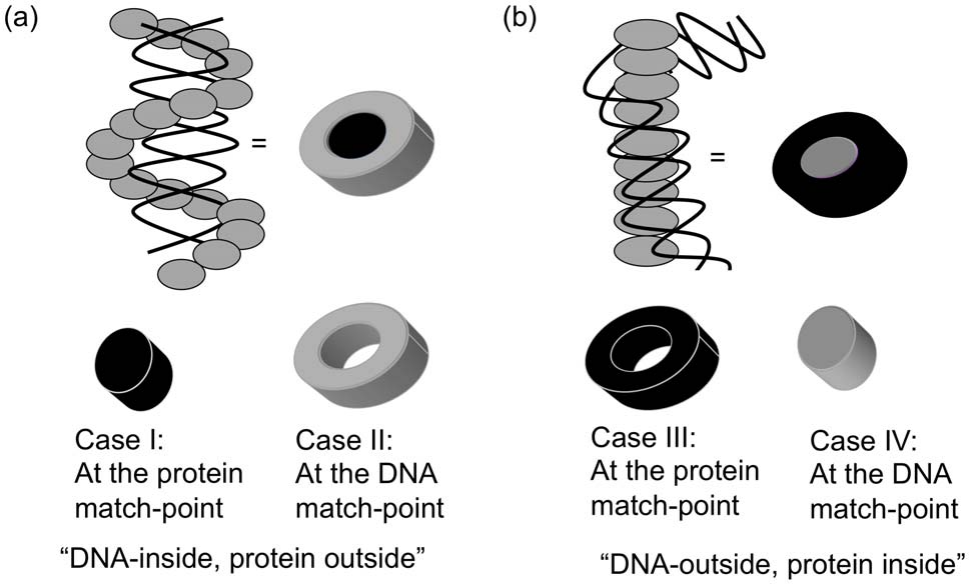
**Hypothetical three-dimensional organizations of the partition assembly.** Two topologically alternative scenarios can be proposed: (a) a “DNA inside, protein outside” model or (b) a “DNA outside, protein inside” model. The DNA is shown as black line, proteins are shown as grey beads. These models can be described as composite cylinders for low-resolution solution scattering experiments. Anticipated shapes at the protein and the DNA match-point for both models (as narrower or hollow cylinders) are shown as cartoons (case I–IV). The handedness and scale are arbitrary. We note that solution scattering cannot distinguish between the left- and the right-handed senses.

The neutron scattering contrast variation data can aid in differentiating between two topologically distinct scenarios in a low-resolution sense: (a) the “DNA-inside, protein-outside” model and (b) the “DNA-outside, protein-inside” model (Figure 2a–b). The previous successes of contrast variation in identifying the correct topology for several nucleoprotein assemblies [36]–[39] prompted us to apply this technique to elucidate the organization of the partition assembly.

**Figure 3:**
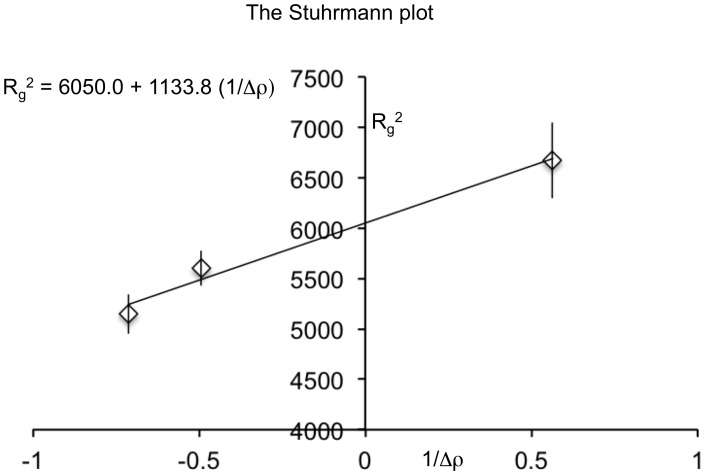
The Stuhrmann plot (R_g_^2^ *versus* Δρ ^−**1**^ **, Δρ in 10^10^ cm** ^−**2**^ **, R_g_ in Å) obtained from the SANS dataset of the tbParB-D120 assembly.** The real space radii of gyration derived from the corresponding pair distribution functions were used for the calculation of Stuhrmann plot. A straight-line fitted to the data (R^2^ = 0.98) is shown in black.

To determine the internal topology of the partition assembly, we analyzed the Stuhrmann plot (Figure 3) [40]–[41];
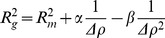
where Δρ is the contrast, R_m_ is the radius of gyration of the entire particle at infinite contrast and the α and β are two contrast-independent parameters. The above reduces to a linear equation when β equals 0. A positive slope of the Stuhrmann plot (α >0) indicated that on average, the DNA, which has a higher scattering length density than protein, is located towards the peripheral region of the rod (Figure 3). A linear Stuhrmann plot (β = 0) suggested a two-component system with coincident centers of mass [41], which is consistent with a “DNA outside, protein inside” filament model with two co-axial polymers (Figure 2). Although we have a limited number of data points, the slope of the Stuhrmann plot clearly supports a “DNA outside, protein inside” organization of the partition assembly.

### A Reduced Cross-sectional Size Near the DNA Match Point Supports a “DNA Outside, Protein Inside” Model

In order to further ascertain a “DNA outside, protein inside” model, we compared the cross-sectional sizes of the partition assembly derived from the SANS datasets at different % of D_2_O. At around ∼65–70% D_2_O, which is the so-called DNA match-point, the scattering contribution from DNA component of a protein-DNA assembly will effectively disappear or be “matched out” by the solvent. A “DNA-inside” model will behave like a hollow cylinder near the DNA match point (Figure 2a). Contrariwise, the size of a “DNA-outside” model near the DNA-match point will shrink due to the lack of any scattering contribution from the outer DNA-occupied regions (Figure 2b).

**Figure 4:**
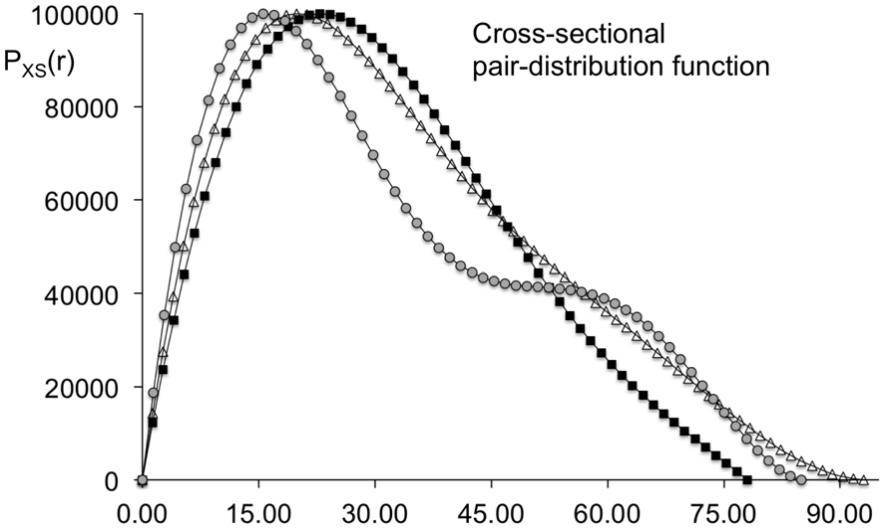
The comparisons of cross-sectional sizes of the tbParB-D120. The cross-sectional pair-distribution functions (P_XS_(r) *versus* the pair-wise distance r in Å) for the 12.0% (open triangle), 85.5% (grey circle) and 73.5% (black square) D_2_O datasets suggests a narrowing of the cross-sectional diameter near the DNA-match point. The P_XS_(r) functions were scaled to an equal maximal height for visualization purpose.

We compared the cross-sectional pair distribution functions (P_xs_(r), calculated using the option 4 in GNOM, [42]–[43]) of tbParB-D120 SANS datasets (Figure 4). The cross-sectional maximal diameters obtained from the P_xs_(r) are 93 Å, 78 Å and 85 Å for the “12.0% D_2_O”, “73.5% D_2_O” and the “85.5% D_2_O” datasets respectively. Changes in the shapes of the P_xs_(r) functions as well as the relative magnitudes of the maximal diameters are consistent with the expected shrinkage of the cross-sectional size near the DNA match-point (“73.5% D_2_O”) in a “DNA outside” model. A 15 Å change in the maximal cross-sectional size between the 73.5% and the 12.0% dataset suggests that the DNA is loosely wound around a protein core.

### Linear Mass Density Analysis

**Figure 5:**
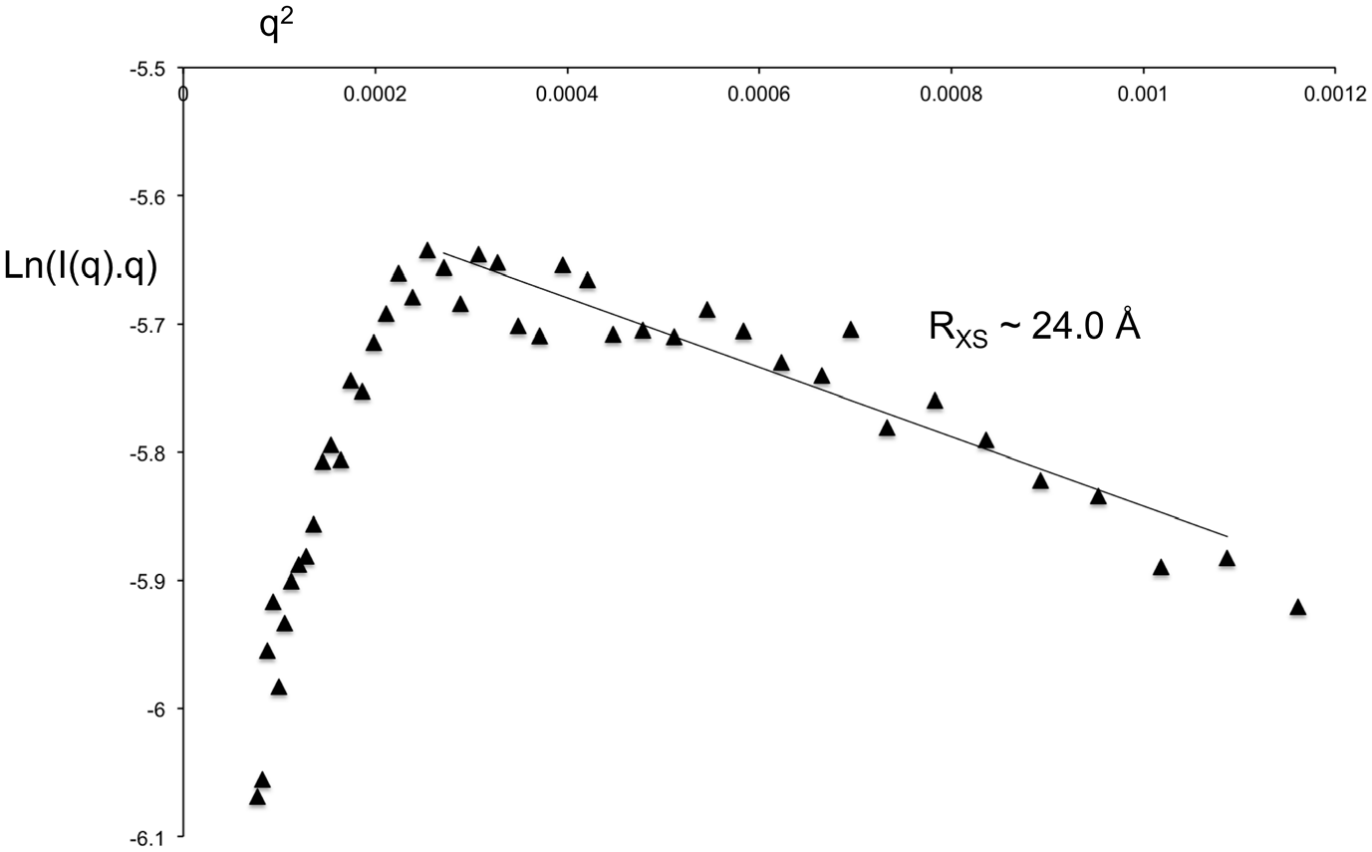
The modified Guinier plot (ln(q.I(q)) *versus* q^2^, I in cm ^−**1**^ **, q in Å** ^−**1**^ **) of the “73.5% D_2_O” dataset.** The R_XS_ and mass/length of the protein segment were obtained from the slope and the intercept of this plot.

Solution scattering data allows an estimation of linear mass density or the mass per unit length of a filament [35], [44]. The intercept (Figure 5) of the modified Guinier plot of the “73.5% D_2_O” dataset provided an estimate of the mass per unit length (M/L in Da/Å) of the protein segment of the tbParB-D120 assembly at 1.9 mg/ml protein concentration.
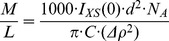
where I_xs_(0) is the intercept of the rod-like Guinier plot in cm^−1^, d is the density (1.35 g/cm^3^), N_A_ is the Avogadro number, C is the concentration in g/l and Δρ is the excess scattering in cm^−2^ units. Mass density of the protein component of the tbParB-D120 particle was estimated to be ∼3988 Da/Å at 73.5% D_2_O, which roughly corresponds to about a protein dimer *per* 20 Å of axial rise. Earlier, we estimated the largest dimension of a tbParB dimer bound to a 22-meric *parS* DNA to be ∼92 Å [27]. The averaged shape of tbParB-*parS* obtained from the *ab initio* shape computations showed that the protein dimer is approximately aligned along the long-axis of DNA [27]. More than 4-fold (20 Å/92 Å) reduction in the projected length of tbParB dimer on the long axis of tbParB-D120 is consistent with protein-induced formation of a DNA super-helix.

### Comparison of the mycobacterial chromosomal partition assembly with the other plasmid-based partition assemblies

The homologs and functional analogs of ParB, such as type I SopB and type II ParR, are generally sequentially and/or structurally diverse [22]. However, formation of a higher order partition assembly between a centromere binding protein, and the centromere region appears to be a common feature in all cases [22]. The crystal structure of a truncated ParR with the centromeric DNA from the plasmid pSK41 showed a large DNA super-helix formation surrounding a protein interior [45]. Pitch and diameter of this super-helix are ∼24 nm and ∼18 nm, respectively. The TEM images revealed a ring-like shape of the pB171 ParR-*parC* (equivalent to ParB-*parS*), with a dimension of 15–20 nm [46]. The crystal structure showed a helical array of ParR in the absence of bound DNA, indicating that the observed, ring-like ParR-*parC* could be a two-dimensional projection of helical organization in the TEM micrograph [46]. A similar super-helical DNA organization was suggested recently for the type III TubR-*tubC* partition assembly [47]. On the other hand, a more extended model of partition assembly that does not involve any DNA wrapping was proposed based on the analyses of SopB-DNA crystal structures [48]. Thus, the internal organization of the mycobacterial partition assembly is similar to some, but not all, of its plasmid-based functional analogs.

## Discussion

We report a set of experiment-derived structural parameters describing the mycobacterial partition assembly, such as the cross-sectional radii of gyration and linear mass density. The neutron scattering H/D contrast variation data presented here is consistent with a “DNA outside, protein inside” mode of the partition assembly organization, which is reminiscent of the DNA-histone complex [37]–[39]. Unlike many previous structural studies on plasmid-based partition assemblies, our data are obtained from a chromosomal partition assembly formed between a DNA with a *parS*-surrounding region and the entire, non-truncated ParB in the solution phase. To the best of our knowledge, this is the first description of the higher order organization of a chromosomal partition assembly, which is a starting point to explore the structural biology of how it recruits different interaction partners, such as ParA, origin tethering factor DivIVA and the SMC proteins, for various biological purposes.

We previously showed that the DNA induces a drastic compaction in the tbParB, which is hypothesized to be required for the higher order partition assembly formation [26]. Our current data suggests a more complex, mutual induced-fit model of the tbParB-DNA interaction. In this model, DNA induces the compaction and polymerization of ParB [26], which in turn induces DNA to form a super-helical array in the outer rim of the protein polymer. Thus, our new data do not support the testable “DNA inside” organization suggested by us earlier [27]. Although our current data do not unambiguously define the geometric parameters of the proposed helical organization, it provides strong evidence that the tbParB organizes a segment of DNA surrounding the *parS* centromere by wrapping it around a protein interior.

Although both plasmid-based SopB and chromosomal ParB belong to the type I group of segrosomes, the organization of the mycobacterial chromosomal partition assembly is intriguingly similar to that of the helical type II ParR-*parC* partition assembly rather than the proposed model of extended SopB-DNA assembly [45]–[48]. The helical structure of the ParR-DNA assembly seemingly assists in capturing the ParM filament for segregation [45]. While several groups reported nucleotide-induced formations of ParA filaments *in vitro*, the existence of such a ParA filament *in vivo* is still a matter of debate [49]–[51]. Therefore, whether ParA is captured by utilizing a ParR-*parC*-like mechanism, or by other mechanism, such as by encircling the DNA-coated outer rim of the mycobacterial partition assembly, remains to be determined.

The DNA wrapping by ParB is particularly significant in the light of recent result that the *parS*-proximal region forms a very compact structure extending over ∼100kb in *C. crescentus* [21]. This *parS* site was shown to be necessary for the maintenance of global genome orientation within the cell [21]. A role of the DNA condensin, which is known to interact with the partition assembly in many bacteria [15]–[17], was suggested in the formation of this compact DNA structure of unknown nature [21]. We showed that the chromosomal tbParB, which shares significant sequence similarity with the *Caulobacter* ParB, wraps a *parS*-containing DNA segment *in vitro* in the absence of an additional DNA condensing agent. We suggest that the ParB-induced initial organization prepares the *parS*-encompassing chromosomal segment for the additional large-scale condensation by DNA condensins and the interactions with DivIVA and ParA in the cellular milieu, using yet to be determined mechanisms.

## Supporting Information

Figure S1 (TIF)
