# Correction: The Internal Organization of Mycobacterial Partition Assembly: Does the DNA Wrap a Protein Core?

**DOI:** 10.1371/annotation/3e3f4f53-4492-4c6e-8426-364bd585b9b8

**Published:** 2013-09-17

**Authors:** Shuo Qian, Rebecca Dean, Volker S. Urban, Barnali N. Chaudhuri

As the result of an error that was introduced during the production process in the Materials and Methods section, there was an error in the equation contained in the 'Solution Neutron Scattering (SANS) with H/D-contrast Variation' section. The correct equation is: ∆λ/λ= 0.15.

