# Supplementary figures and images for: The Internal Organization of Mycobacterial Partition Assembly: Does the DNA Wrap a Protein Core?
Source: PLoS One. 2012 Dec 20;7(12):e52690. doi: 10.1371/journal.pone.0052690 (PMC3527565; doi:10.1371/journal.pone.0052690)

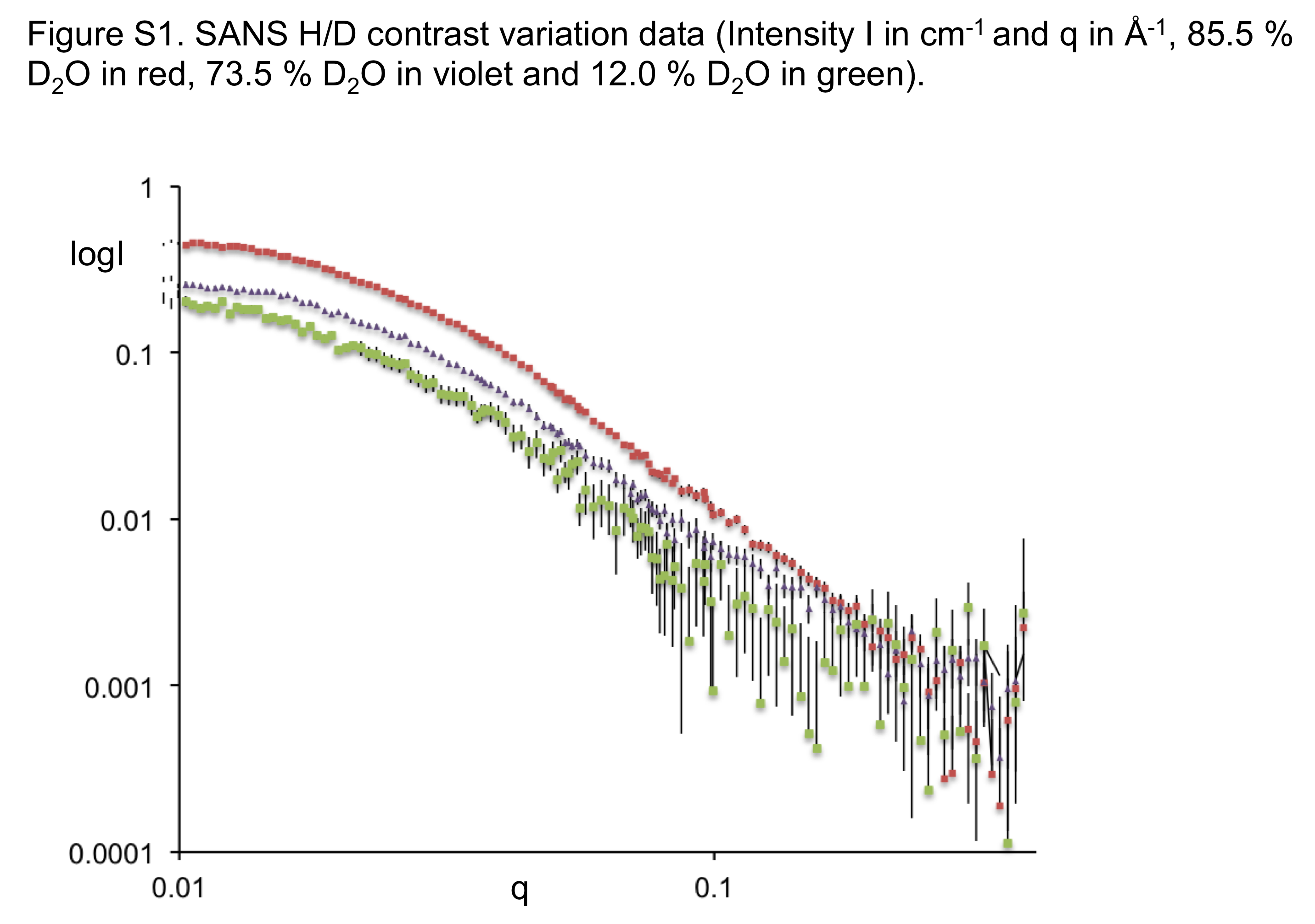

Supplement: Figure S1 — (TIF) [file pone.0052690.s001.tif]
